# Effect of toothpastes containing different NaF concentrations or a SnF2/NaF combination on root dentine erosive lesions, *in vitro*

**DOI:** 10.4317/jced.53047

**Published:** 2016-12-01

**Authors:** Iliana Diamanti, Haroula Koletsi-Kounari, Eleni Mamai-Homata

**Affiliations:** 1 Visiting Postdoctoral Researcher, Department of Preventive and Community Dentistry, Dental School, National & Kapodistrian University of Athens, Athens, Greece; 2 Associate Professor, Department of Preventive and Community Dentistry, Dental School, National & Kapodistrian University of Athens, Athens, Greece

## Abstract

**Background:**

Fluoride toothpastes presumably offer some protection against acid erosion. However, uncertainty exists towards fluoride’s efficacy relatively to the concentration and the type of chemical compound used. This *in vitro* study evaluated the relative efficacy of toothpastes containing sodium fluoride in different concentrations or a stabilized stannous fluoride/sodium fluoride system on root dentine erosion.

**Material and Methods:**

Bovine dentin specimens were allocated into four groups (n=10): control (no F), 1450ppm F (as NaF), 5000ppm F (as NaF) and 1450ppm F (1100ppm as stabilized SnF2 and 350ppm as NaF)/sodium hexamethaphosphate. The specimens were submitted to 6 daily cycles of erosion (0.3% v/v citric acid, pH=3.2, 20 min) and remineralization (~22h), interspersed by 2-min immersions in 1:3 w/v of dentifrice/distilled water slurries. Subsequently, they were subjected to a 24-h acid resistance test (0.3% v/v citric acid, pH=3.2) without any further treatments. Surface loss was quantified by contact profilometry. Data were analysed through one-way ANOVA and Bonferroni’s tests (*p*≤0.05).

**Results:**

In both experiments, all fluoride groups, showed significantly less tissue loss compared to the control (*p*<0.001-*p*=0.018). During erosion cycling, no significant differences were found among the fluoride groups. During the acid resistance test, the 5000ppm F toothpaste produced significantly superior effect than both 1450ppm F products (*p*=0.010, (*p*<0.001), which performed similarly.

**Conclusions:**

Under less aggressive erosive conditions, fluoride toothpastes did not differ in their ability to protect dentine surfaces. However, in severely erosive environment, the 5000ppm F toothpaste performed superiorly to the other tested products.

** Key words:**Dentine, sodium fluoride, stannous fluoride, tooth erosion, toothpaste, contact profilometry.

## Introduction

As dentine is often exposed even at relatively early stages of tooth erosive lesion formation (1), it is therefore an important target tissue for implementing erosion – inhibiting strategies. *In vitro*, dentine erosive lesion formation process results in the development of a surface layer of fully demineralized, acid – insoluble collagenous matrix (2), however, clinically, it is not yet clear whether and to what extent this structure is retained in the oral cavity (3).

Evidence suggests that erosion-inhibiting strategies should focus at increasing surface resistance of dentine tissue against subsequent erosive challenges (4). The application of solutions of conventional monovalent fluorides (e.g. sodium fluoride) or polyvalent metal fluorides (stannous fluoride) has demonstrated some erosion-suppressing ability *in vitro* and *in situ* (5,6) although their effectiveness may be related to the presence of the demineralized organic matrix zone (7).

Few studies have examined fluoridated toothpastes’ effectiveness on suppression of erosive demineralization or erosive wear on dentine. Conventional NaF formulations (1100-1500ppm F) were shown to provide 25%-32% more protection against erosion or erosion/abrasion insults compared to non – fluoridated controls *in vitro* and *in situ* (8-10) however, no such effect was observed in other *in vitro* experiments (9,11-14). In view of these limited effects, highly concentrated NaF dentifrices (5000ppm F) were tested for erosion-inhibiting purposes˙ however, they seemed to offer no added benefit compared to conventional NaF products (1100ppm F), *in vitro* and *in situ* (8,12). Stannous fluoride toothpastes have been also tested for their erosion – suppression capability and dentifrices containing 1100-1450ppm F (as SnF2) demonstrated up to 64% more reduction in dentine tissue loss compa-red to non – fluoridated products, *in vitro* and *in situ* (13,15,16).

In recent years, a toothpaste containing stabilized SnF2 (1100ppm F) and NaF (350ppm F) compounds combined with sodium hexametaphosphate, an inorganic polymer claimed to create a protective layer onto the tooth surface, thus blocking susceptible sites from erosive acid insults (17) has been available commercially. Concerning the inconsistent findings about the efficacy of conventional and highly concentrated NaF toothpastes against erosive challenge on dentine, as well as the preliminary evidence that Sn – containing toothpastes may hold promising anti - erosive potential for dentine tissue, it appears reasonable to compare the performance of dentifrices containing various NaF concentrations as well as the stabilized SnF2 technology, during relatively severe erosive attacks, resulting in dentine oral exposure.

Therefore, the aim of the present *in vitro* study was to assess the ability of three commercially available toothpastes, containing 5000ppm F (1.1% NaF), 1450ppm F (0.319% NaF) and a 1450ppm F [1100ppm as stabilized SnF2 (0.454%) and 350ppm as NaF (0.077%)]/sodium hexamethaphosphate - based formula respectively, to inhibit bovine root dentine demineralization by a dietary acid. For that purpose, an erosion cycling model was initially applied and was followed by secondary exposure of treated areas in acid, in order to assess the ”acquired acid resistance” of dentine *in vitro*.

## Material and Methods

A total of 40 bovine root dentine slabs were used, which were allocated to four toothpaste groups of ten slabs each. Toothpaste slurries (1:3 w/v in distilled water) were applied on sound root dentine slabs, during exposure to six daily demineralization/remineralization cycles, leading to formation of erosive lesions. The pH values of the tested dentifrices were obtained with a pH-electrode (Ross Ultra® Glass Combination, Thermo Scientific, Orion, U.S.A) using the respective slurries and the toothpastes were either calcium carbonate- or silica-based (non-fluoridated control and fluoridated products, respectively). The following toothpaste slurries were applied: (a) non-fluoridated (control; pH=9.16), (b) 1450ppm F (as NaF; pH=7.30), (c) 5000ppm F (as NaF; pH=8.16) and (d) 1450ppm F (1100ppm as stabilized SnF2 and 350ppm as NaF)/sodium hexamethaphosphate (pH=5.48).

-Erosive solution: The erosive solution (ER) contained citric acid that was prepared to a concentration of 0.3% v/v with the pH adjusted to 3.2 using NaOH buffer, following previously published protocols (18).

-Remineralizing solution: Τhe remineralizing solution (RM) contained 3mM Ca, 1.8mM P (derived from 17mM tricalcium phosphate) in 1% carboxy-methyl-cellulose and was adjusted to pH 7.0.

-Preparation of dentine slabs: Sections approximately 3×5×3mm, were cut from previously unexposed to the oral environment buccal and lingual root surfaces of permanent bovine lower incisors, extracted from bovines that were sacrificed at 2.5-3.0 years of age and stored in thymol/tap water solution at 4°C. The sections were prepared with the use of a water-cooled low-speed diamond disk mounted in a sectioning machine (Bronwill Scientific, Rochester N.Y, U.S.A). Each root provided four sections and each section was allocated to each of the four toothpaste groups. The slabs were mounted with sticky wax on Plexiglass blocks and were progressively polished until the tubule orifices were uniformly exposed at each experimental surface. The polishing procedure was performed with wetted 600-, 1200-, 2000- and 4000-grit silicon carbide papers (P600, P1200 and P2000 Wetor-dryTM 734, 3M Bracknell, United Kingdom and FEPA P# 4000, Struers, Ballerup, Denmark) and subsequently the polished slabs were examined microscopically (20x), to ensure the quality of polishing. One third of each flat, polished dentine surface was covered by two layers of acid-resistant nail varnish to maintain a reference surface for tissue loss determination, leaving exposed two thirds of the surface which was subjected to the erosive challenge.

**Figure 1:**
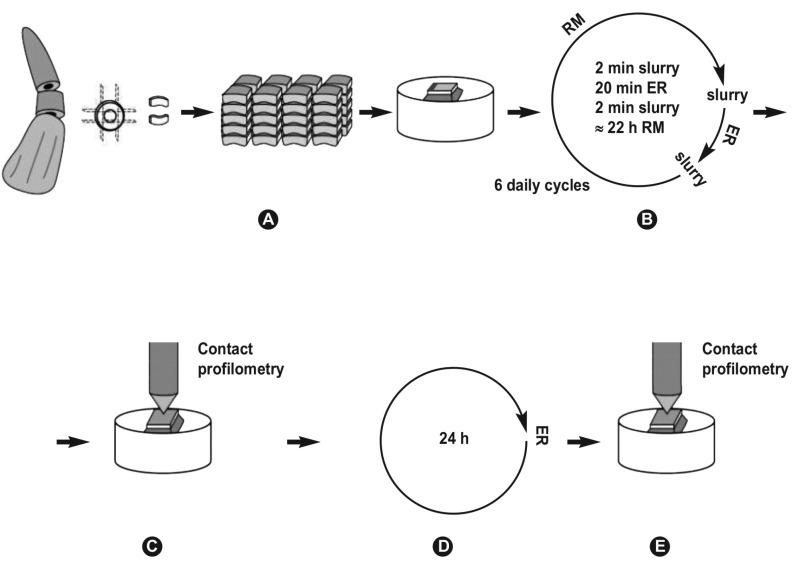
A) Bovine root dentine slabs’ (3mm×5mm×3mm) preparation. B) Erosive challenge based on a 6-day pH-cycling model interspersed to dentifrice slurry applications. C) The specimens were processed for contact profilometry. D) The surfaces were subjected to a 24-h acid resistance test, without any further treatments. E) The specimens were processed for final contact profilometry measurements.

-Experimental procedure: Initially, dentine slabs were subjected to six daily ER/RM cycles. Each daily cycle comprised a 20-min erosive challenge period and two 2-min toothpaste slurry treatment periods, before and after immersion in the unstirred acidic solution (10 ml/slab). All erosion and intervention procedures were performed at room temperature (approximately 20°C); in between, the slabs were kept in remineralizing solution (20 ml/slab, renewed at every change), at 37°C (approximately 22 h per day). All slabs were rinsed with distilled water for 15 s before and after any erosive/remineralizing solution change or toothpaste slurry application and were wiped dry with soft tissue paper. After the end of the 6-day erosion cycling period, all specimens were assessed for residual acid resistance with a 24-h acid resistance test (ART), without any further treatments. In figure 1, the described experimental protocol is presented schematically.

**Figure 2:**
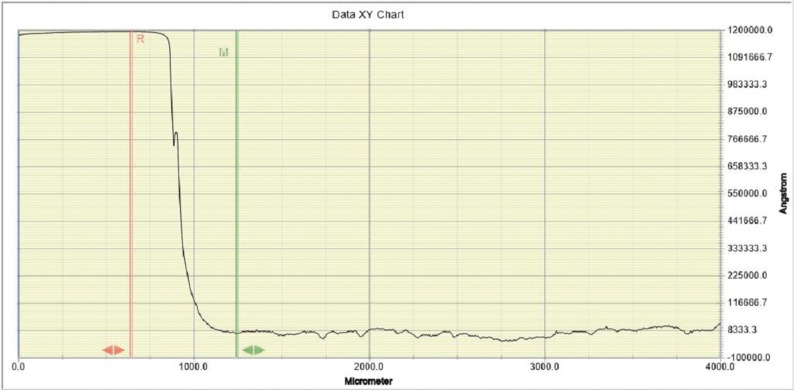
Data plot of a typical profilometric scan of an eroded dentin surface. Measurement of the step height between the reference (sound) and the treated (eroded) surface was performed, with the use of respective reference (red) and measurement (green) cursors.Erosion depth was determined by measuring the vertical distance from the highest point of the reference surface (sound dentine) to the lowest point of the eroded surface profile within the first 300μm from the erosive step.

-Contact profilometry measurements: Dentine surface loss (μm) was quantitatively determined by a contact profilometer (Dektak® 150 Stylus Profiler, Veeco Instruments Inc, US) after the end of the erosion cycling period as well as after the 24-h acid challenge. For each profilometry measurement the nail varnish was carefully removed using a scalpel and the specimens were allowed to dry in ambient air (≈24h), in order to minimize the possible influence that the shrinkage of the organic content might cause to the profilometrical measurements (19). Stylus force calibration (measurement range: 1-15mg) as well as vertical calibration (step height measurement range: 6.5-524μm) against certified values were performed before lesion depth measurements. The vertical accuracy of the system was ±0.5% and the software used for the analysis was Dektak® 150 software running under Windows XP. The radius of the diamond stylus was 12.5μm and the horizontal resolution of the system was 0.250μm/sample. The stylus moved laterally from the reference to the exposed area across the long axis of the rectangular shaped surface with a weight of 5.0mg and a scan length of 4.0mm during 60 s. Three parallel profile scans were performed for each sample at intervals of 0.75mm. Data plot of a typical profilometric scan of an eroded dentine surface is shown in figure 2. Step height measurement procedure was standardized with the use of the appropriate software for the analysis, so as to achieve high reproducibility of the obtained values. Specifically,each profilometric line scan was adjusted to the x-axis by levelling accordingly the eroded part of the trace. Lesion depth was determined by measuring the vertical distance from the highest point of the reference surface (sound dentine) to the lowest point of the eroded surface profile within the first 300μm from the erosive step, as was indicated in previously published studies (20). For each sample a mean was calculated from the values obtained from the three line scans. During measurements,the observers were not blinded with respect to the type of toothpaste treatment that each specimen had been exposed to.

-Statistical analysis: A total sample size of 40 specimens (10 per experimental group) was calculated to provide 95% statistical power to detect an effect size of (minimum) 0.70 at 5% significance level, by using G*Power version 3.1.9.2 software tool for statistical power analysis (21). The outcome variable was the maximum erosion depth (μm) from the original (sound) dentine surface that was profilometrically determined twice, at the end of the erosion cycling and of the acid resistance test periods. Mean and standard deviation (SD) values of erosion depth per toothpaste group were calculated. Statistical procedures were performed using the Statistical Package for the Social Sciences (SPSS 17.0, Chicago, IL, USA) for Windows. There was no significant deviation of erosion depth values distribution from the Gaussian curve in each group (Kolmogorov-Smirnov test: *p*=0.200) and furthermore, variances did not differ significantly among the toothpaste groups (Levene’s test of homogeneity of variances: erosion cycling experiment: *p*=0.098, erosion resistance test: *p*=0.515). Therefore, one-way analysis of variance (ANOVA) was performed for the comparisons between the groups. Post - ANOVA contrasts were performed using a Bonferroni correction for pair-wise comparisons. Values of *p*≤0.05 were accepted as being statistically significant.

## Results

 Table 1 summarizes erosion depth (μm) data obtained after the end of the erosion cycling procedure as well as after the acid resistance test, showing mean and standard deviation by toothpaste type. It can be seen that in both experiments, all fluoridated groups presented lower surface loss values compared to the non-fluoridated (control) group. The observed effect sizes were calculated to be 1.46 and 1.22 for the erosion cycling experiment and the acid resistance test,respectively. The statistical analysis ( Table 2) revealed that, during erosion cycling, all test products showed a significant positive effect compared to the control, in the order of 33%-37% (*p*<0.001 each). Among the fluoride groups, during erosion cycling, no significant differences were found (*p*=1.000). During the acid resistance test, all fluoridated groups exhibited significantly less surface loss compared to the control group, in the order of 10%-25% (*p*=0.001, *p*<0.001, *p*=0.018). Furthermore, the 5000ppm F (as NaF) toothpaste inhibited surface loss significantly more effectively than the 1450ppm F (as NaF) (*p*=0.010) and the stabilized SnF2 (1100ppm F)/NaF (350ppm F)/sodium hexamethaphosphate products (*p*<0.001), while the two 1450ppm F groups presented similar acid resistance behavior (*p*=1.000).

**Table 1:**
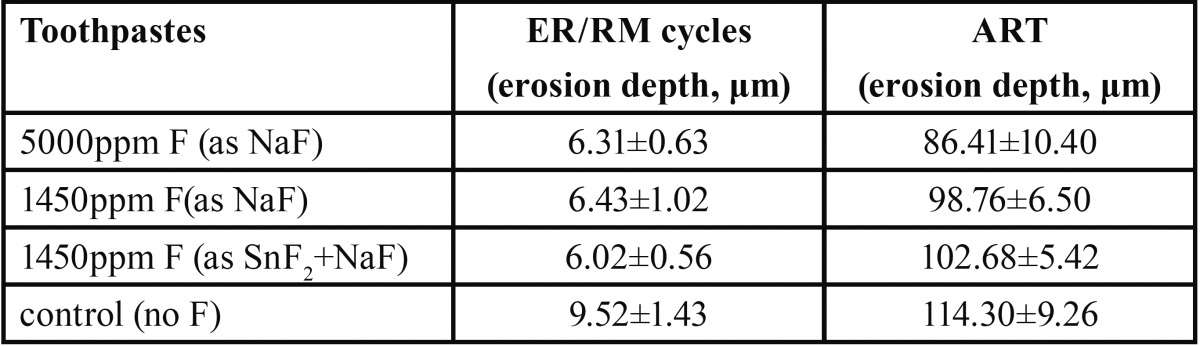
Mean values and standard deviations of erosion depth measurements (μm) by toothpaste type, after the erosion/remineralization cycles (ER/RM) and the acid resistance test (ART).

**Table 2:**
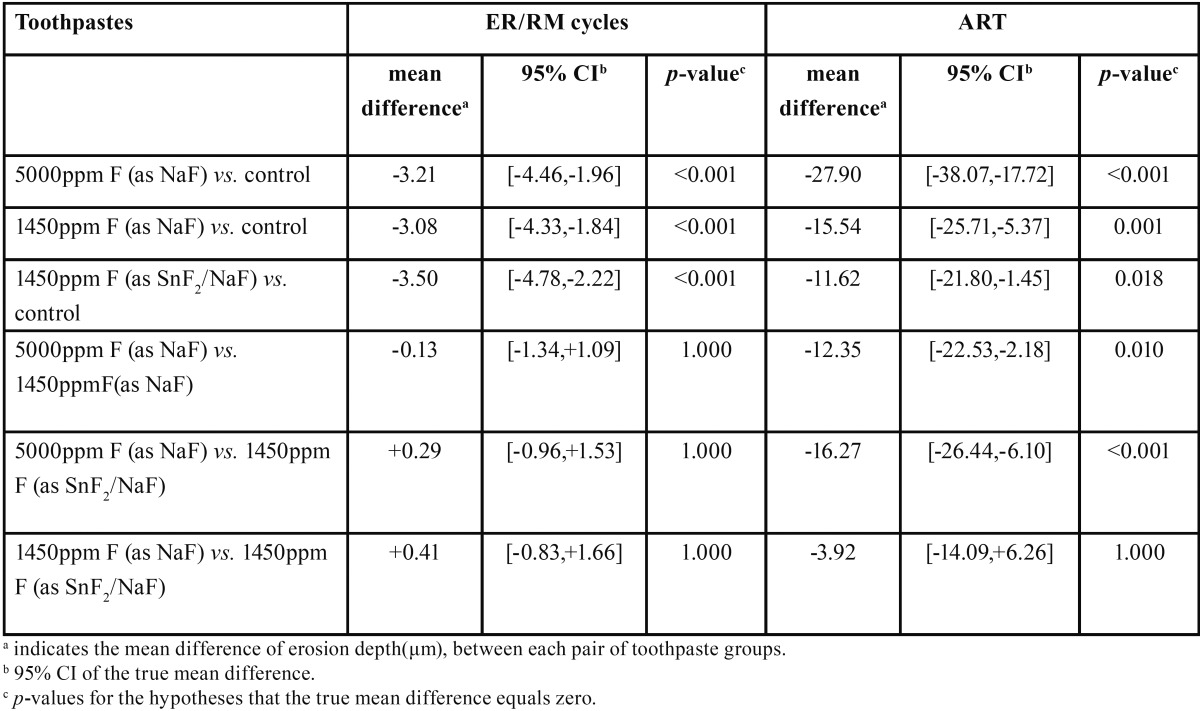
Post – ANOVA pairwise comparisons between the toothpaste groups after the erosion/remineralization cycles (ER/RM) and the acid resistance test (ART), based on Bonferroni correction of Type I error.

## Discussion

The present *in vitro* study assessed the relative preventive effect of toothpastes containing different concentrations of NaF or a stabilized SnF2/NaF/sodium hexamethaphosphate - based system, on surface loss of bovine root dentine lesions created by implementing a 6-day erosive cycling protocol, followed by testing of the “acquired resistance” of treated mineralized tissue under a continuous, prolonged erosive challenge. The experimental model that was utilized aimed to simulate a severely erosive clinical environment and was designed so as to provide conditions balanced in a way to identify differences among the various compounds under test.

Contact profilometry is a method widely applied for assessing hard tissue loss in relation to a non-treated reference area. However, in eroded dentine, the persistence of a superficial,completely demineralized collagenous matrix zone could interfere with the stylus tip movement across the lesion surface, so that, depending on the moisture level of the tissue, the latter may not reach the demineralization front (22). To partially overcome this limitation, the specimens were allowed to sufficiently dry in ambient air prior to the profilometry measurements in order to stabilize the hydration status of the tissue, thus minimizing the possible measurement inaccuracy caused by dimensional changes during shrinkage of the organic content (19). Accordingly, the present dentin surface loss results should be interpreted taking into account the existence of a dehydrated layer of organic material in the experimental area.

At the end of the erosion cycling period all fluoridated groups exhibited significantly less dentine surface loss compared to the non-fluoridated control group and this effect persisted even after 24h of continuous immersion of the specimens in the erosive solution. These results are in agreement with observations derived from other purely erosion experimental studies, where conventional NaF toothpastes (1100-1500ppm F) and a SnF2 dentifrice (1400ppm F) demonstrated superior effectiveness in inhibiting dentine erosion compared to non-fluoridated products (8,10,16). However, in certain *in vitro* settings, no additional benefit related to NaF incorporation in the toothpaste could be observed (9,11).

The potential of topical applications of sodium fluoride to inhibit dental erosive demineralization is attributed to the formation of a CaF2 – like layer which is assumed to act as a physical barrier, providing some additional mineral to be dissolved during an acid challenge before the underlying hard tissue is attacked (10). CaF2 layer may also act as a mineral reservoir, which, by increasing its dissolution rate during an acid attack induces acid buffering, enhances fluoride adsorption over the crystal surfaces or accelerates surface precipitation of more acid-resistant F - containing apatites and therefore the overall stability of the hard tissue will tend to increase (23,24).

Considering the mode of action of stannous fluoride on dentine, in case the superficial layer of fully demineralized organic matrix that develops is preserved, it has been suggested to be related to tin’s attraction by phosphorus, some potentially negative charged collagen groups and/or certain phosphoproteins and retention in the demineralized organic portion of the tissue to some extent, but mainly, as tin possess higher affinity for mineral, to its diffusion through this structure and accumulation in the underlying mineralized dentine (25).

Presumably, the above chemical mechanisms provide a reasonable explanation for the observed superiority in erosion-protective capability between the dentifrices containing SnF2 and/or NaF and the non-fluoridated product. However it has been stated that the preventive effect of fluoride compounds on dentine erosion appears to be highly dependent on the retention of the demineralized surface layer of organic material (7,26) and this effect is probably related to its buffering capacity leading to the establishment of less aggressive erosive conditions at the demineralization front (7).

In the current study, no significant difference in surface loss was found between the groups fluoridated with NaF only at different concentrations, at the end of the erosion cycling period. These results are in agreement with other *in vitro* and *in situ* experiments, where the application of 5000ppm F (as NaF) in the form of commercial toothpaste or experimental liquid toothpaste following certain erosion cycling protocols, offered no added benefit against dentine erosion and erosive wear compared to 1100-1450ppm F (as NaF) products (8,12). However, during the 24h acid resistance test, the 5000ppm F toothpaste was observed to inhibit erosive demineralization significantly more effectively than the 1450ppm F dentifrice. As the beneficial effect of sodium fluoride is associated with the formation of a CaF2 – like material, it is likely that by application of more highly concentrated fluoride agents the amount of this precipitate might be increased (27) leading to more crystallite surface coverage and analogous reduction of the rate of mineral dissolution (28,29). Therefore, it is possible that in the erosion cycling experiment, the erosive effect was relatively small to allow the detection of significant differences among the dentifrices containing different sodium fluoride concentrations in contrast, the more severe erosive environment that was established during the acid resistance test, enabled the demonstration of the protective effect of sodium fluoride compound in terms of a dose – response relationship.

Finally, in the present experimental setting, no significant difference was found between the 1450ppm F (as NaF) and the 1100ppm F (as stabilized SnF2)+350ppm F (as NaF) groups. Furthermore, the latter, likewise to the former, demonstrated inferior performance during the acid resistance test compared to the 5000ppm F toothpaste group. Commercial versions of 1100ppm F (as SnF2)/350ppm F (as NaF) and of 1500ppm F (as NaF) toothpaste were shown to be able to similarly inhibit dentine erosive wear, *in vitro* (13), however, *in situ*, a NaF toothpaste (1100ppm F) was more effective than a SnF2 product (1100ppm F), but the former was less abrasive (30). *In vitro* studies showed that, in the presence of the demineralized organic matrix, if equal concentrations of F ion in solution were applied, the type of fluoride compound (NaF or SnF2) was not crucial for the level of erosion-inhibition, whereas, removal of the matrix reduced markedly the efficacy only of the NaF compound (26). However, other *in vitro* experiments did not demonstrate a relationship between the presence of the collagenous matrix and the relative erosion- inhibiting potential of various fluoride compounds (11).

Considering the variation in results among the different studies, it appears likely that the variable experimental parameters that were used, including the variable consistencies of the toothpaste formulations that were applied, even if the type and concentrations of the active ingredients were similar, may have had a significant impact on their effects. Therefore, it appears difficult to generalize conclusions for types of active agents in toothpaste formulations (e.g. NaF–, SnF2– toothpastes) based on testing of single or only few products from each type.

In the present experiment, a purely erosion rather than an erosion/abrasion model was used. It has been suggested that mineral loss may not be significantly affected by clinically relevant abrasion insults (11). However, abrasive forces could hamper the process of mineral precipitation or partly remove surface precipitates, essential for the anti – erosive effect (31,32). Therefore, considering that no straightforward interplay exists between the various experimental parameters participating in erosion studies, we cannot discard the possibility that the inclusion of toothbrush abrasion forces might have influenced the relative protective potential of the tested toothpastes.

In conclusion, under the experimental conditions of the present study the findings showed that, a sodium fluoride toothpaste with high fluoride concentration (5000ppm F), under severe erosive conditions, appears to inhibit dentine surface loss more effectively than a conventional sodium fluoride dentifrice (1450ppm F), or a dentifrice containing a stabilized stannous fluoride (1100ppm F)/sodium fluoride (350ppm F)/sodium hexamethaphosphate system.
